# Targeted modulation of *IGFL2‐AS1* reveals its translational potential in cervical adenocarcinoma

**DOI:** 10.1002/1878-0261.70217

**Published:** 2026-02-03

**Authors:** Ricardo Cesar Cintra, Lucca Paolo Hsu Helmich, Daniel Rodrigues de Bastos, Laura Sichero, Patrícia Savio de Araujo‐Souza, Fabio Passetti, Luisa Lina Villa

**Affiliations:** ^1^ Center for Translational Research in Oncology, Instituto do Cancer do Estado de São Paulo ICESP Hospital das Clinicas da Faculdade de Medicina da Universidade de São Paulo FMUSP HC São Paulo Brazil; ^2^ Comprehensive Center for Precision Oncology Universidade de São Paulo São Paulo Brazil; ^3^ Department of Radiology and Oncology Faculdade de Medicina da Universidade de São Paulo São Paulo Brazil; ^4^ Faculdade de Medicina da Universidade de São Paulo São Paulo Brazil; ^5^ Facultad de Ciencias Médicas Universidad Privada del Este Ciudad del Este Paraguay; ^6^ Laboratory of Immunogenetics and Histocompatibility, Department of Genetics Universidade Federal do Paraná (UFPR) Curitiba Brazil; ^7^ Instituto Carlos Chagas, FIOCRUZ Paraná Brazil

**Keywords:** adenocarcinoma, cervical cancer, CRISPR/Cas9, *IGFL2‐AS1*, prognostic biomarker

## Abstract

Cervical cancer remains a leading cause of mortality among women, particularly in low‐ and middle‐income countries. Despite distinct prognoses and clinical outcomes between its main histological subtypes, squamous cell carcinoma (SCC) and adenocarcinoma (ADC), current treatment regimens remain largely similar, creating an urgent need for targeted therapeutic strategies based on molecular distinctions. To address this gap, the long noncoding RNA *IGFL2‐AS1* was investigated as a potential prognostic biomarker and therapeutic target in cervical adenocarcinoma. A translational approach was employed that integrated patient transcriptome data, *in silico* analysis from public databases, and *in vitro* validation. Using CRISPR/dCas9 technology, *IGFL2‐AS1* expression was modulated in HeLa (ADC) and SiHa (SCC) cell lines to assess its impact on cellular characteristics associated with tumorigenesis. *In silico* analysis revealed that *IGFL2‐AS1* expression was significantly reduced in ADC compared to SCC, and its low expression was consistently linked to poorer ADC prognosis and decreased patient survival. Notably, overexpression of *IGFL2‐AS1* in HeLa cells significantly reduced cell proliferation, migration, clonogenic survival, and enhanced sensitivity to cisplatin and doxorubicin. Conversely, *IGFL2‐AS1* repression in SiHa cells yielded no significant phenotypic changes, suggesting a context‐dependent mechanism. *IGFL2‐AS1* is identified as a histological subtype‐specific prognostic biomarker and promising therapeutic target for cervical adenocarcinoma.

## Graphical abstract

Cervical adenocarcinoma patients face worse outcomes than squamous cell carcinoma counterparts despite similar treatment. The identification of *IGFL2‐AS1*'s differential expression provides a molecular basis for distinguishing these histotypes, paving the way for personalized therapies and improved survival in vulnerable populations globally.

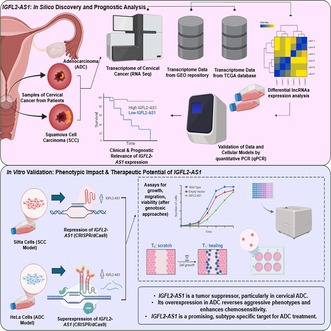

**Abbreviations**
5‐AzadC: 5‐Aza‐2′‐deoxycytidine
ADC: adenocarcinoma
ATCC: American Type Culture Collection
CC: cervical cancer
ceRNAs: competing endogenous RNAs
CI: confidence interval
CR: closing rate
CRISPR/dCas9: clustered regularly interspaced short palindromic repeats/dead Cas9
DMSO: dimethyl sulfoxide
EMT: epithelial–mesenchymal transition
FBS: fetal bovine serum
FIGO: International Federation of Gynecology and Obstetrics
GAPDH: glyceraldehyde 3‐phosphate dehydrogenase
GDC: genomic data commons
HCI: high‐content imaging
HNRNPC: heterogeneous nuclear ribonucleoprotein C
HPV: human papillomavirus
HR: hazard ratio
HR‐HPV: high‐risk human papillomavirus
ICESP: Instituto do Câncer do Estado de São Paulo
lncRNA: long noncoding RNA
MEM: minimum essential medium
miRNAs: microRNAs
MOI: multiplicity of infection
PAM: protospacer adjacent motif
PE: plating efficiency
qPCR: quantitative polymerase chain reaction
qRT‐PCR: quantitative reverse transcription‐polymerase chain reaction
SAM‐Cas9: synergistic activation mediator‐Cas9
SCC: squamous cell carcinoma
SEM: standard error of the mean
SF: surviving fraction
sgRNA: single guide RNA
TCGA: The Cancer Genome Atlas
TP: paclitaxel and platinum
TPM: transcripts per million
TSA: trichostatin A
TSS: transcription start site
WT: wild type

## 1 Introduction

In 2022, it was estimated that cervical cancer (CC) affected 660 000 women worldwide, resulting in approximately 350 000 deaths [1]. It is well‐established that this type of cancer is primarily caused by persistent infections with high‐risk human papillomavirus (HR‐HPV), which are transmitted mainly through sexual contact [2]. Availability of screening programs, early detection and treatment, and prophylactic vaccines makes this cancer largely preventable. However, these strategies are more accessible in developed countries. Consequently, about 90% of CC cases and related deaths occur in low‐ and middle‐income countries, where limited access to preventive healthcare strategies leads to late diagnoses and, consequently, higher mortality rates [3].

The two main histological subtypes of CC are squamous cell carcinoma (SCC) and adenocarcinoma (ADC). Interestingly, while HPV type 16 is the predominant high‐risk type contributing to both histological subtypes, HPV types 18 and 45 are also frequently associated with an increased risk of adenocarcinoma [4]. Although ADC is less frequent than SCC, a relative increase in ADC cases has been reported over the years, primarily attributed to the inherent challenges in detecting glandular lesions at pre‐invasive stages during gynecological screening [5]. Despite clear differences in molecular pathogenesis, histological appearance, and clinical behavior between SCC and ADC, current treatment guidelines largely recommend similar therapeutic approaches for both, which disadvantages ADC patients who generally have poorer clinical outcomes [6, 7].

Typically, ADCs exhibit greater invasiveness into adjacent tissues, higher recurrence rates, distant metastases, and resistance to treatment [8]. Given that differences between SCC and ADC histological subtypes have been explored regarding protein‐coding genes, there is also a growing recognition of a gene expression regulatory network based on noncoding RNAs. Unlike protein‐coding mRNAs, the expression of lncRNAs appears to be more restricted, often specific to certain cell types or even particular ontogenetic stages of differentiation [9, 10]. Long noncoding RNAs (lncRNAs) are attributed with a range of functions, including cis/transcriptional regulation, nuclear domain organization, protein interaction, and regulation of other RNA molecules, for instance, by acting as competing endogenous RNAs (ceRNAs) that sponge microRNAs (miRNAs) [11]. Due to their involvement in diverse cellular processes, many lncRNAs have already established roles in CC, and some have even been used as diagnostic and prognostic markers [12]. In this context, our group is investigating the expression of a set of lncRNAs that can distinguish between SCC and ADC. Building upon this, our previous work identified distinct transcriptional regulatory networks and differentially expressed lncRNAs that characterize these histological subtypes [13].

Over the past years, accumulated evidence has identified *IGFL2‐AS1* as a potential oncogene in colon, renal, tongue, breast, and gastric cancer. It has been attributed functions related to autophagy, migration, invasion, chemo/radioresistance, and immune modulation of the tumor microenvironment, including export through extracellular vesicles [14, 15, 16, 17, 18, 19]. Intriguingly, no studies have yet explored the role of this lncRNA in CC.

In this study, we initially explored differentially expressed lncRNA data from CC samples available in the TCGA database and identified *IGFL2‐AS1* as a potential biomarker candidate capable of distinguishing between SCC and ADC. Once we observed that low expression of *IGFL2‐AS1* was associated with poor prognosis, we explored the therapeutic potential of fine‐tuning its expression using a modified CRISPR/Cas9 system. This approach enabled us to specifically target *IGFL2‐AS1* in cell lines representing SCC and ADC, and further evaluate its impact on phenotype and treatment response behavior. Moreover, we investigated the effects of epigenetic modulators on *IGFL2‐AS1* expression patterns within these distinct histological contexts. *In vitro* experiments demonstrated that inducing *IGFL2‐AS1* expression in the ADC model significantly reduced cell proliferation, migration, and clonogenic survival (both before and after irradiation), while also sensitizing them to genotoxic agents.

## 2 Methods

### 2.1 Patient cohort transcriptome and public database *in silico* analysis

Our initial identification of *IGFL2‐AS1* and other lncRNAs (TINCR, CALML3‐AS1, DSG1‐AS1, LINC02381, and LINC01833) as differentially expressed between SCC and ADC cervical subtypes was derived from the transcriptomic analysis of a cohort of women treated at the Instituto do Câncer do Estado de São Paulo (ICESP), as previously detailed [13]. Building upon this finding, we further investigated the expression profile and prognostic significance of *IGFL2‐AS1* using public databases and bioinformatics resources. GENT2 is a bioinformatics tool that enables differential gene expression analysis and prognostic evaluation based on data from control and tumor tissues, as well as cell lines. The information available in GENT2 was extracted from the TCGA and GEO databases. In this context, ‘control’ refers to normal adjacent tissue samples or samples from healthy individuals, as annotated within these public repositories [20]. A keyword search for *IGFL2‐AS1* allowed us to obtain data on RNA expression across various tissue types, with a particular focus on the uterine cervix tissue. The gene expression file corresponding to the GPL570 platform (HG‐U133_Plus_2) was retrieved for analysis. Comparisons between control and tumor tissues were conducted using the Mann–Whitney statistical test, ensuring a robust assessment of the observed expression differences. The cBioPortal platform (www.cbioportal.org) was utilized to access publicly available data provided by The Cancer Genome Atlas (TCGA) and the Genomic Data Commons (GDC). The analysis focused on normalized RNA expression in transcripts per million (TPM) and adjusted z‐scores for the *IGFL2‐AS1*, *TINCR*, *CALML3‐AS1*, *DSG1‐AS1*, *LINC02381*, and *LINC01833* genes. Expression data for 309 CC tumor samples were obtained from the TCGA‐CESC cohort. Four cases with unavailable expression information were excluded, resulting in a final cohort of 305 eligible cases for analysis [21, 22, 23]. Next, the expression data were integrated with clinicopathological information to explore potential correlations between *IGFL2‐AS1* expression and histological subtype. To ensure a quantitative assessment of the *IGFL2‐AS1* expression profile, data normality was initially checked. Depending on the distribution characteristics, appropriate statistical tests, such as the *t*‐test or Mann–Whitney test, were applied in the analysis of variables. The median gene expression values were calculated, allowing for the stratification of patients into two groups: low expression (defined as the given value being lower or equal than the median) and high expression (defined as the given value being higher than the median). Survival analysis was conducted using the Kaplan–Meier method and the log‐rank test. Additionally, Cox regression analyses, both univariate and multivariate, were performed to calculate hazard ratios (HR), accompanied by 95% confidence intervals (95% CI). The variables considered in the univariate analysis included patient age, tumor grade, HPV status (HPV16 vs. HPV18), histological subtype (SCC vs. ADC), FIGO stage, and *IGFL2‐AS1* expression levels. Variables with a *P*‐value < 0.05 in the univariate analysis were subsequently included into the multivariate Cox regression model to identify independent prognostic factors. Statistical significance for all analyses was defined as a *P*‐value ≤0.05.

### 2.2 Cell culture

CC cell lines derived from SCC and ADC, specifically SiHa (RRID: CVCL_0032) (HPV16; ATCC® HTB‐35™) and HeLa (RRID: CVCL_0030) (HPV18; ATCC® CCL‐2™), respectively, were obtained from the American Type Culture Collection (ATCC, Manassas, VA, USA). The selection of these cell lines was strategic for studying the role of *IGFL2‐AS1* in cervical cancer subtypes. The HeLa cell line was chosen as it is the only commercially available and well‐characterized primary cervical adenocarcinoma cell line. For the squamous cell carcinoma (SCC) model, the SiHa cell line was selected after an initial screening of several SCC cell lines, including C33A, SW756, and CaSki. SiHa exhibited the highest basal *IGFL2‐AS1* expression among the SCC cell lines evaluated (data not shown), which was crucial for maximizing the impact of the planned gene repression experiments. These cells were cultured in Minimum Essential Medium (MEM) (Gibco™, Invitrogen, Carlsbad, CA, USA) supplemented with 10% fetal bovine serum (Gibco™, Invitrogen), 100 μg·mL^−1^ streptomycin, and 100 μg·mL^−1^ penicillin. For all experiments, parental SiHa and HeLa cells were routinely maintained at low passage numbers (typically between passages 3 and 10) to ensure phenotypic stability and minimize genetic drift. Cell cultures were maintained in incubators under standardized conditions of 5% CO_2_ at 37 °C. Following stable transduction and selection, the CRISPR/dCas9‐modified HeLa (*IGFL2‐AS1* ^+^) and SiHa (*IGFL2‐AS1* ^−^) cell lines were cryopreserved at early passages. For all functional assays described herein, aliquots of these modified cell lines were thawed and expanded, with experiments performed within a maximum of 5 passages after thawing to ensure consistency. For lentivirus production, HEK293T (RRID: CVCL_0063) (ATCC CLR 3216™) cells were used and cultured under the same medium and conditions as above. All human cell lines used in this study have been authenticated within the last 3 years. Authentication was performed annually by the institutional service using a standardized method (ANSI/ATCC ASN‐0002‐2011) based on Short Tandem Repeat (STR) analysis. The obtained profiles were compared with reference databases from internationally recognized repositories (DSMZ, ATCC, JCRB, and RIKEN). Furthermore, we confirm that all experiments were performed with mycoplasma‐free cells.

### 2.3 Treatment with 5‐aza‐2′‐deoxycytidine (5‐AzadC) and trichostatin a (TSA)

To investigate the potential role of epigenetic mechanisms in regulating *IGFL2‐AS1* expression across distinct CC histological subtypes, we used the epigenetic modulating agents 5‐Aza‐2′‐deoxycytidine (5‐AzadC), a DNA methyltransferase inhibitor, and Trichostatin A (TSA), a histone deacetylase inhibitor. Optimal non‐cytotoxic concentrations for both compounds were determined similarly: cells (1 × 10^4^ per well) were seeded in 6‐well plates and treated with varying concentrations of each drug for 7 days. The cell number was determined by counting and compared to the vehicle‐treated control wells. For 5‐AzadC (Sigma‐Aldrich, St. Louis, MO, USA), the vehicle was DMSO, and a concentration of 10 μm was selected for both cell lines as the highest dose tested that did not cause a significant reduction in cell number compared to DMSO controls. The medium was replaced daily throughout the 5‐AzadC treatment period due to its instability in aqueous media. For TSA (Sigma‐Aldrich), the vehicle was ethanol. A non‐cytotoxic concentration of 100 nm was selected for individual TSA treatments. For combined treatments, 75 nm TSA was used in conjunction with 7.5 μm 5‐AzadC. All experiments involving these drugs were performed at least three times independently.

### 2.4 RNA isolation and reverse transcription‐polymerase chain reaction (qRT‐PCR)

Total RNA was isolated by the direct application of 1 mL of TRIzol (Invitrogen) to the cells, according to the manufacturer's protocol. RNA samples were quantified, and their purity concerning proteins and organic compounds contamination was confirmed by 260/280 and 260/230 nm ratios using the NanoDrop2000® spectrophotometer (Thermo Fisher Scientific, Waltham, MA, USA). RNA integrity was verified using the TapeStation2100® (Agilent Technologies Inc., Santa Clara, CA, USA) and inferred based on the RNA Integrity Number (RIN). Two micrograms of total RNA from each sample were treated with RNase‐free DNase Set® (Qiagen, Hilden, Germany) and subjected to reverse transcription using SuperScript™ Vilo™ (Life Technologies, Carlsbad, CA, USA). Specific primer pairs for the *IGFL2‐AS1* gene (RefSeq NR_135234.1) and *GAPDH* (RefSeq NR_152150.2) were designed using NCBI Primer‐BLAST (www.ncbi.nlm.nih.gov), analyzed with OligoAnalyzer (IDT, Coralville, IA, USA), and synthesized by Thermo Fisher Scientific [24, 25]. The primer sequences (from 5′ to 3′) were as follows: human *IGFL2‐AS1* forward: AGCCAACAAGAATGAAGAAGTGG, human *IGFL2‐AS1* reverse: TTCACACCTCTTCCGCTGTC, and human *GAPDH* forward: GACTGTGGTCATGAGTCCTCCC, human *GAPDH* reverse: CAAGATCATCAGCAATGCCTCC. qRT‐PCR assays were performed using Go Taq® qPCR Master Mix (Promega Corp., Madison, WI, USA) on the ABI Prism 7500 instrument (Applied Biosystems, Foster City, CA, USA), and relative quantification was verified by the delta–delta CT method. Prior to performing the experimental assays, the limit of detection and quantification for *IGFL2‐AS1* and *GAPDH* were rigorously validated to ensure reliable and reproducible measurements, particularly given the generally lower expression of lncRNAs. All Ct values for *IGFL2‐AS1* reported in this study were below 33, confirming robust detection and quantification within the linear range of the assay.

### 2.5 CRISPR/dCas9‐mediated modulation of *IGFL2‐AS1* expression

CRISPR/dCas9‐mediated transcriptional modulation was used to alter *IGFL2‐AS1* expression in CC cell lines. The CRISPR/dCas9‐VP64‐p65 system was employed for transcriptional activation in HeLa cells (ADC model) [26], while the CRISPR/dCas9‐KRAB‐MeCP2 system was used for transcriptional repression in SiHa cells (SCC model) [27].

### 2.6 sgRNA design

The *IGFL2‐AS1* gene promoter region was identified using the eukaryotic ncRNA promoter database (https://epd.expasy.org) [29]. Two single guide RNA (sgRNA) sequences were designed within the −200 and −20 bp regions relative to the transcription start site using CRISPOR (https://crispor.tefor.net), with 20 bp‐NGG PAM optimized for SpCas9 to minimize off‐target effects [30]. The sgRNA sequences targeting the *IGFL2‐AS1* promoter region, designed for both CRISPR/dCas9 activation (SAM‐Cas9) and repression (KRAB‐MeCP2), are shown in Fig. S1A, along with their positions relative to the transcription start site (TSS) (−107 and −161 bp). The specific sequences of these sgRNAs and the adaptor regions are detailed in Fig. S1B.

### 2.7 Cloning of expression vectors and lentivirus production

The plasmids lenti_dCas9‐KRAB‐MeCP2 (catalog #122205), lenti_dCas9‐VP64_Blast (catalog #61425), lenti_MS2‐P65‐HSF1_Hygro (catalog #61426), and lenti_sgRNA(MS2)_zeo (catalog #61427) were obtained from Addgene (Watertown, MA, USA). The cloning of the sgRNA into lenti_sgRNA(MS2)_zeo was performed according to the protocol detailed by Konnermann et al. [26]. The insertion of sgRNAs into the plasmids was verified by Sanger sequencing. Lentivirus production was conducted in HEK293T cells (ATCC CLR 3216™) using the Mission™ Lentiviral Pack Mix reagent (Sigma‐Aldrich) according to the manufacturer's instructions. Lentivirus titration was performed using a qPCR method [31].

### 2.8 Generation of cell lines with modulation of *IGFL2‐AS1* expression

The concentration of antibiotics for selection of transduced cells was determined by cytotoxicity assay, establishing concentrations of 6 μg·mL^−1^ blasticidin, 250 μg·mL^−1^ hygromycin, and 100 μg·mL^−1^ zeocin, during 4 days for the first two antibiotics and 10 days for the third, respectively. For transduction, 1 × 10^4^ cells were seeded in 6‐well plates 24 h prior to transduction. A multiplicity of infection (MOI) of 0.5 was used in culture medium containing 8 μg·mL^−1^ Polybrene (Sigma‐Aldrich) for 48 h, followed by a selection period with the respective antibiotics. Expression modulation was verified by qRT‐PCR, and for analytical comparisons, groups were established including the parental cell lines (wild type), cell lines transduced with the empty vector, or the experimental vector, designated as HeLa ^WT^, HeLa ^∅^, HeLa ^*IGFL2‐AS1*+^, SiHa ^WT^, SiHa ^∅^, SiHa ^*IGFL2‐AS1‐*^.

### 2.9 Cell proliferation assay

A total of 5 × 10^3^ cells per well were seeded in 24‐well plates for each cell population. Cell proliferation was assessed every 48 h in technical triplicates using a hemocytometer. Each experiment was conducted at least three times. Cell number records were transferred to a spreadsheet and analyzed using graphpad prism 5 for Windows (GraphPad Software, San Diego, CA, USA).

### 2.10 Clonogenic assay

On the day before irradiation, 2 × 10^5^ cells were seeded in 6‐well plates (9.6 cm^2^) for each cell population. One plate for each cell population was left unirradiated and used as the control; these control cells were processed in the same manner as their irradiated counterparts. Twenty‐four hours after irradiation, cells from both the irradiated and unirradiated (control) plates were plated in technical triplicates at the following densities: 0.7 × 10^3^ cells per well for HeLa and 1 × 10^3^ cells per well for SiHa. Cells were maintained in culture until the colonies were sufficiently large to be visible for macroscopic counting. This typically took approximately 9 days for HeLa and 14 days for SiHa. Subsequently, colonies were fixed with 1 mL of 4% formaldehyde for 15 min, washed with PBS, and stained with 0.1% crystal violet for 20 min. After a final PBS wash to remove excess stain, the plates were allowed to dry for 1 day. Finally, the colonies were manually counted, and the number of colonies in each condition was used to calculate the surviving fraction. The Plating Efficiency (PE) for each condition was determined as (number of colonies formed/number of cells plated) × 100. The Surviving Fraction (SF) was then calculated as the PE of treated cells divided by the PE of unirradiated control cells. Each experiment was conducted as at least three independent biological replicates.

### 2.11 Wound healing assay

A total of 2 × 10^5^ HeLa cells per well and 3 × 10^5^ SiHa cells per well were seeded in 12‐well plates (3.6 cm^2^) in quadruplicate and cultured in MEM supplemented with 10% FBS until reaching 90–100% confluence. Subsequently, the cells were treated with 10 μm mitomycin C for 2 h, and a scratch was introduced using a 200 μL pipette tip. Images of HeLa cells were captured at 0‐, 24‐, and 36‐h post‐scratch, while images of SiHa cells were captured at 0‐, 24‐, and 48‐h post‐scratch. At each time point, a perpendicular line to the scratch was marked with a pen, and the same region was photographed at each time point. Each independent experiment was performed at least three times. The scratch area was quantified using imagej software and a plugin [32]. To calculate the closing rate (CR), the following formula was used:

$$\mathrm{CR}=1-\frac{\text{area}\;\mathrm{at}\;\text{time}\;X}{\text{starting area}}*100.$$

### 2.12 Cell viability after genotoxic treatment

Cell viability analysis was performed using the AlamarBlue™ reagent (Invitrogen™). Before the assay, IC50 values for cisplatin and doxorubicin were determined for both cell lines, with parameters verified at cancerrxgene.org [28, 33]. Thus, we defined 20 μm cisplatin and 0.4 μm doxorubicin for HeLa, and 19 μm cisplatin and 0.4 μm doxorubicin for SiHa cells. One day before drug administration, 1 × 10^3^ cells from each line were seeded in octoplicates in 96‐well plates. After 72 h of drug administration, the medium with the respective drugs was removed, and 100 μL of culture medium containing 10% Alamar Blue reagent was added to each well. After 90 min of incubation, fluorescence data were collected using the GloMax® Discover Microplate Reader (Promega) and analyzed with graphpad prism 5 for Windows (GraphPad Software, La Jolla, CA, USA). Each experiment was performed at least three independent times.

### 2.13 Caspase 3/7 activity analysis and apoptosis induction

Caspase 3/7 activity and apoptosis induction were assessed using a fluorogenic assay that relies on active caspases cleaving a substrate to release a quantifiable fluorescent signal. High‐content imaging (HCI) was employed for both qualitative and quantitative assessment. HeLa cells (adenocarcinoma model), including *IGFL2‐AS1* overexpressing cells, were seeded at 5 × 10^3^ cells per well in triplicate in black Advanced TC 96‐well microplates (Greiner Bio‐One, Kremsmünster, Austria, #655986). After cisplatin treatment, cells were incubated for 36 and 48 h, corresponding to half and two‐thirds of the AlamarBlue assay incubation. Medium was aspirated, and 100 μL per well of CellEvent Caspase‐3/7 Green Detection Reagent (Thermo Fisher Scientific, #C10423) at 2 μm final concentration was added. Cell nuclei were co‐stained with Hoechst‐33 342 (1 : 3000; Thermo Fisher Scientific, #H3570). Plates were incubated at 37 °C for 30 min in the dark before image acquisition. Images were obtained using the MetaXpress High‐Content Image Acquisition & Analysis Software (Molecular Devices, San Jose, CA, USA), capturing nine random fields per well at 20× magnification. Automated cell quantification identified nuclei based on Hoechst‐33342 fluorescence. Caspase‐3/7 activity was determined by the CellEvent Caspase‐3/7 reagent fluorescence intensity above local background. Overall cell viability, as presented in the results, was derived from the instrument's output by multiplying the average percentage of adhered cells by the percentage of cells negative for caspase 3/7 staining (live cells), thus inferring cellular viability. Each experiment was performed at least three independent times. Statistical significance was determined by ANOVA with Tukey's *post‐hoc* test.

## 3 Results

### 3.1 *IGFL2‐AS1* expression is significantly reduced in cervical adenocarcinoma and associated with poor prognosis

**Fig. 1:**
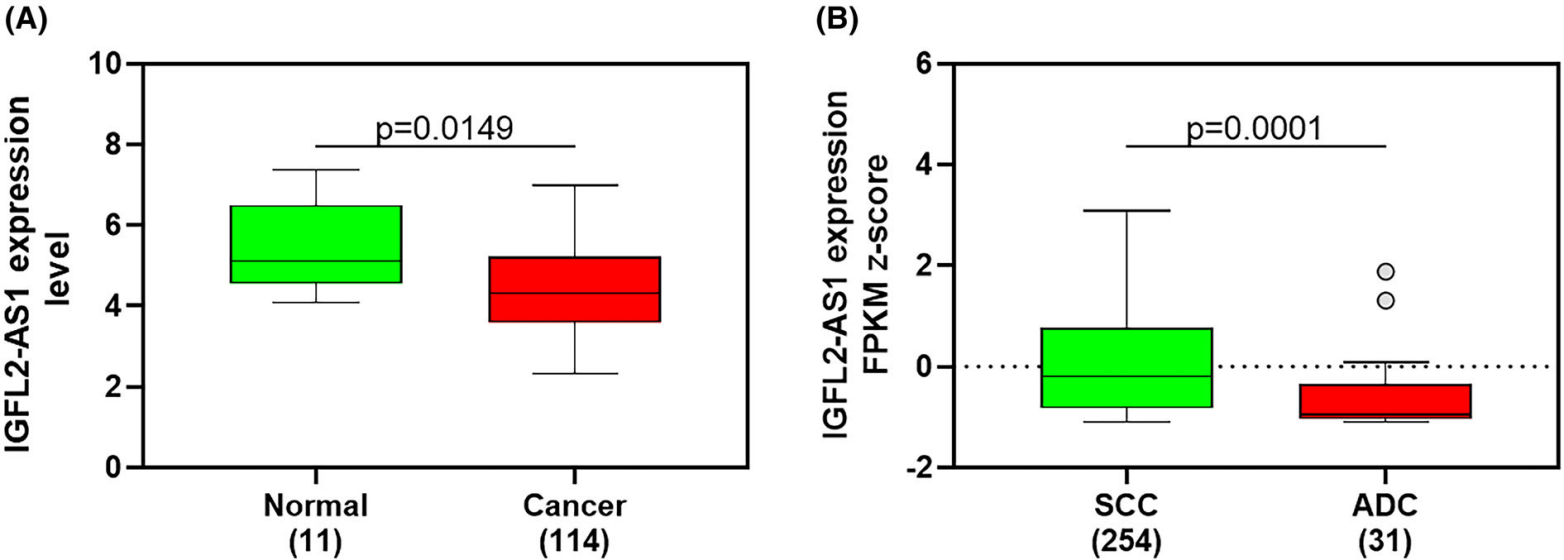
*IGFL2‐AS1* RNA levels in cervical samples from GENT and TCGA data sources by (A) expression profile in control and tumor tissue; (B) histological subtype categorized in SCC and ADC expression profile. The graphs correspond to box‐and‐whisker plots with outliers displayed by the Tukey method, in which the horizontal lines represent the quartiles and the whiskers indicate distribution dispersion. Number of samples: *n* = 11 normal and *n* = 114 cancer in panel A; *n* = 254 SCC and *n* = 31 ADC in panel B. Statistical comparisons were conducted using the Mann–Whitney test. ADC, adenocarcinoma; SCC, squamous cervical carcinoma.

*In silico* analysis, utilizing both GENT2 and The Cancer Genome Atlas (TCGA) databases, revealed significant findings regarding *IGFL2‐AS1* expression in cervical tissues. Notably, *IGFL2‐AS1* expression was significantly reduced in tumor tissues compared to control tissues (*P* = 0.0149) (Fig. 1A). Furthermore, focusing on the histological tumor subtype, *IGFL2‐AS1* expression was significantly lower in ADC compared to SCC (*P* = 0.0001) (Fig. 1B), suggesting a distinct role for *IGFL2‐AS1* in these histological subtypes, with particularly low expression in ADCs. To further explore its potential association with HPV types, Fig. S2A shows that *IGFL2‐AS1* expression was significantly lower in tumors associated with HPV18 compared to HPV16, regardless of the histological subtype (*P* = 0.0245).

**Fig. 2:**
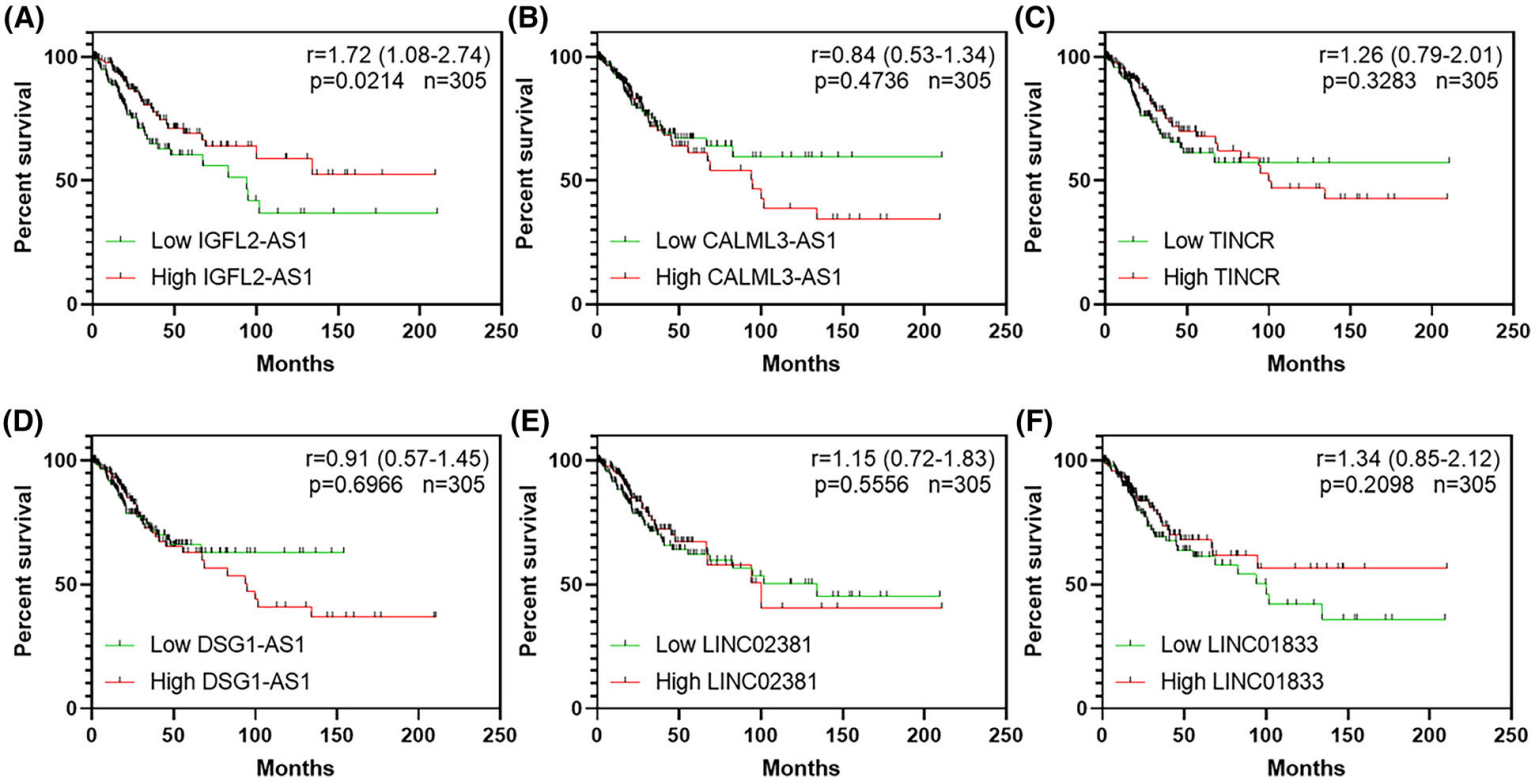
Overall survival of cervical cancer patients according to lncRNA expression regardless of the histological subtype for (A) *IGFL2‐AS1*; (B) *CALML3‐AS1*; (C) *TINCR*; (D) *DSG1‐AS1*; (E) *LINC02381* and (F) *LINC01833*. Survival data were obtained from TCGA and GDC databases. Each Kaplan–Meier curve includes tick marks representing censored observations. Patients were stratified as low or high expression groups based on the median expression level of each analyzed gene. Comparisons between expression groups were performed using the log‐rank statistical test. Hazard Ratios and corresponding Confidence Intervals (CI) were estimated using Cox regression analysis.

The cBioPortal platform (www.cbioportal.org) was used to access public data from The Cancer Genome Atlas (TCGA) and the Genomic Data Commons (GDC). Our analysis focused on normalized RNA expression in transcripts per million (TPM) and adjusted z‐scores for the genes *IGFL2‐AS1*, *CALML3‐AS1*, *TINCR*, *DSG1‐AS1*, *LINC02381*, and *LINC01833*, identified as differentially expressed between SCC and ADC, previously identified by us [13]. Survival analysis using the Kaplan–Meier method revealed that among these lncRNAs, only lower *IGFL2‐AS1* transcript levels showed a statistically significant association with poorer overall survival (Fig. 2A). In contrast, *CALML3‐AS1*, *TINCR, DSG1‐AS1*, *LINC02381*, and *LINC01833* did not exhibit statistically significant associations with overall survival (Fig. 2B–F, respectively). Specifically, lower *IGFL2‐AS1* expression levels were linked to a poorer prognosis, with a hazard ratio (HR) of 1.72 (95% CI: 1.08–2.74) and a *P*‐value of 0.0214 (Fig. 2A). This robust association observed across the 305 samples provided strong justification for focusing on modulating *IGFL2‐AS1* expression in subsequent experimental validation. Importantly, when restricting the survival analysis to adenocarcinoma patients only, Fig. S2B revealed an even more pronounced association, with low *IGFL2‐AS1* levels conferring a significantly worse prognosis compared to high *IGFL2‐AS1* levels (HR = 9.17; 95% CI: 1.05–80.56; *P* = 0.0456).

In the univariate Cox regression analysis, FIGO stage and low *IGFL2‐AS1* expression were statistically significant associated with poorer overall survival (*P* < 0.05). In contrast, age, tumor grade, HPV status, and histological subtype did not show statistically significant associations and were therefore excluded from the multivariate model. In this adjusted model, FIGO stage III/IV remained significantly associated with worse prognosis (HR = 2.72; 95% CI: 1.65–4.48; *P* < 0.0001). Importantly, low *IGFL2‐AS1* expression also retained its prognostic value, emerging as an independent predictor of poor overall survival (HR = 2.06; 95% CI: 1.27–3.33; *P* = 0.0033), thereby reinforcing its potential role as a clinically relevant biomarker in CC (Table 1).

**Table 1:** Overall survival analysis of cervical cancer patients from the TCGA/GDC cohort. ADC, adenocarcinoma; FIGO, International Federation of Gynecology and Obstetrics; SCC, squamous cell carcinoma.

Variables	Univariate		Multivariate	
	HR (95% CI)	*P*‐value	HR (95% CI)	*P*‐value
Age				
≤ 40	1	0.1498		
> 40	1.50 (0.86–2.62)			
Grade				
I/II	1	0.5501		
III/IV	1.08 (0.83–1.40)			
HPV status				
HPV16	1	0.2175		
HPV18	1.79 (0.71–4.49)			
Histological subtype				
ADC	1	0.2175		
SCC	1.17 (0.47–2.93)			
FIGO				
I/II	1	0.0006	1	< 0.0001
III/IV	2.33 (1.44–3.79)		2.72 (1.65–4.48)	
*IGFL2‐AS1*				
High	1	0.0214	1	0.0033
Low	1.72 (1.08–2.74)		2.06 (1.27–3.33)	

### 3.2 Characterization of *IGFL2‐AS1* expression patterns, CRISPR/dCas9‐mediated modulation, and response to epigenetic and genotoxic treatments in cervical cancer cell models

**Fig. 3:**
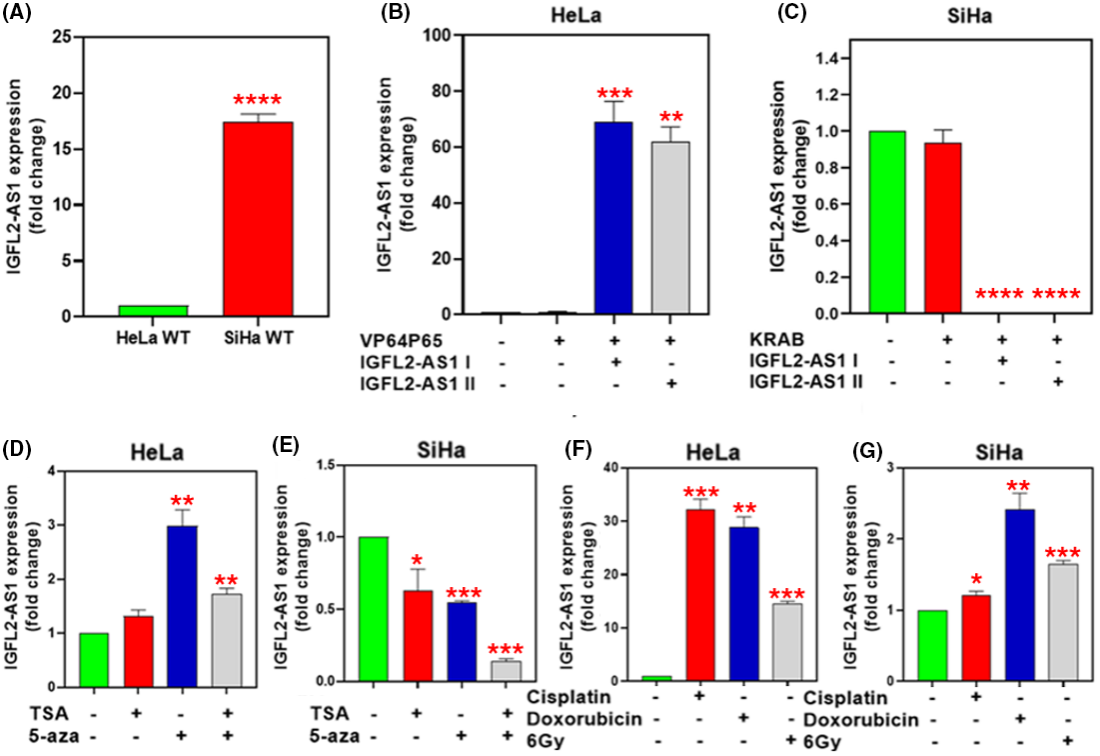
*IGFL2‐AS1* expression patterns and modulation in cervical cancer cell lines by CRISPR/dCas9, epigenetic, chemotherapeutic, and irradiation treatments. All data presented in this figure represent *IGFL2‐AS1* expression levels determined by quantitative RT‐PCR. (A) Baseline *IGFL2‐AS1* expression levels in SiHa and HeLa cell lines. Expression was normalized to *GAPDH* and is shown relative to HeLa cells. (B) Validation of *IGFL2‐AS1* overexpression in HeLa cells. (C) Validation of *IGFL2‐AS1* repression in SiHa cells. (D) Effect of epigenetic treatments (5‐AzadC, TSA, and their combination, 7 days) on *IGFL2‐AS1* expression in HeLa cells. (E) Effect of epigenetic treatments (5‐AzadC, TSA, and their combination, 7 days) on *IGFL2‐AS1* expression in SiHa cells. (F) *IGFL2‐AS1* expression in HeLa cells following treatment with cisplatin, doxorubicin, or irradiation. (G) *IGFL2‐AS1* expression in SiHa cells following treatment with cisplatin, doxorubicin, or irradiation. Fold change is relative to untreated cells. Data represent the mean ± standard error of the mean (SEM) of at least three independent experiments. We analyzed differences among treatment using the Kruskal–Wallis test followed by Dunn's multiple comparison *post‐hoc* test, and the corresponding *P*‐values are report in the figures indicated by *P < 0.05, **P < 0.01, ***P < 0.001, or ****P < 0.0001.

We first examined the baseline *IGFL2‐AS1* expression levels in the selected CC cells SiHa (SCC) and HeLa (ADC). The expression pattern was consistent with our patient sample cohort and the *insilico* analyses, showing higher *IGFL2‐AS1* expression in SiHa cells compared to HeLa cells (Fig. 3A).

The quantitative RT‐qPCR was performed to assess the overexpression and inhibition (Fig. 3B,C, respectively) of *IGFL2‐AS1* in transduced cells using the CRISPR/dCas9 system. While two sgRNAs were initially designed and used for gene modulation, subsequent functional assays were conducted exclusively with the sgRNA that induced the most pronounced and successful modulation of *IGFL2‐AS1* expression. This selection strategy ensured that our analyses were focused on the sgRNA for which the CRISPR/dCas9 system had exerted the greatest impact on target gene expression, thereby maximizing the sensitivity and robustness of our subsequent experimental results.

Furthermore, the effects of epigenetic drug treatments on *IGFL2‐AS1* expression varied significantly between the two cell lines. In HeLa cells, treatment with 5‐AzadC resulted in a nearly threefold increase in *IGFL2‐AS1* expression. While treatment with TSA alone induced a more subtle increase (~ 1.3‐fold), the combination of 5‐AzadC and TSA resulted in an increase of approximately 1.9‐fold, indicating no superior effect compared to 5‐AzadC alone (Fig. 3D). Conversely, in SiHa cells, individual administration of 5‐AzadC led to a ~ 40% reduction in *IGFL2‐AS1* expression, while TSA alone resulted in a ~ 30% reduction. Notably, the combined administration of these epigenetic drugs produced a significant synergistic negative effect, leading to a substantial 90% reduction in *IGFL2‐AS1* expression. This differential responsiveness to epigenetic treatments highlights distinct patterns of epigenetic influence on *IGFL2‐AS1* expression in ADC versus SCC (Fig. 3E).

To determine whether chemotherapeutic agents and irradiation influence *IGFL2‐AS1* expression, cells were subjected to a 48‐h treatment with half the IC50 dose for each respective chemotherapeutic agent and with the LD50 dose of irradiation for both cell lines. The qRT‐PCR analysis revealed differential regulation of *IGFL2‐AS1* expression in HeLa and SiHa cell lines after the indicated treatments. HeLa cells exhibited a significantly greater induction of *IGFL2‐AS1* expression following cisplatin treatment (~ 31‐fold), doxorubicin treatment (~ 27‐fold), or irradiation (~ 10‐fold) (Fig. 3F). In contrast, SiHa cells showed a subtle increase in *IGFL2‐AS1* expression with doxorubicin and irradiation (approximately 2.3‐ and 1.5‐fold, respectively), with no significant change observed after cisplatin treatment (Fig. 3G).

### 3.3 Differential effects of *IGFL2‐AS1* modulation on cell proliferation in cervical cancer models

**Fig. 4:**
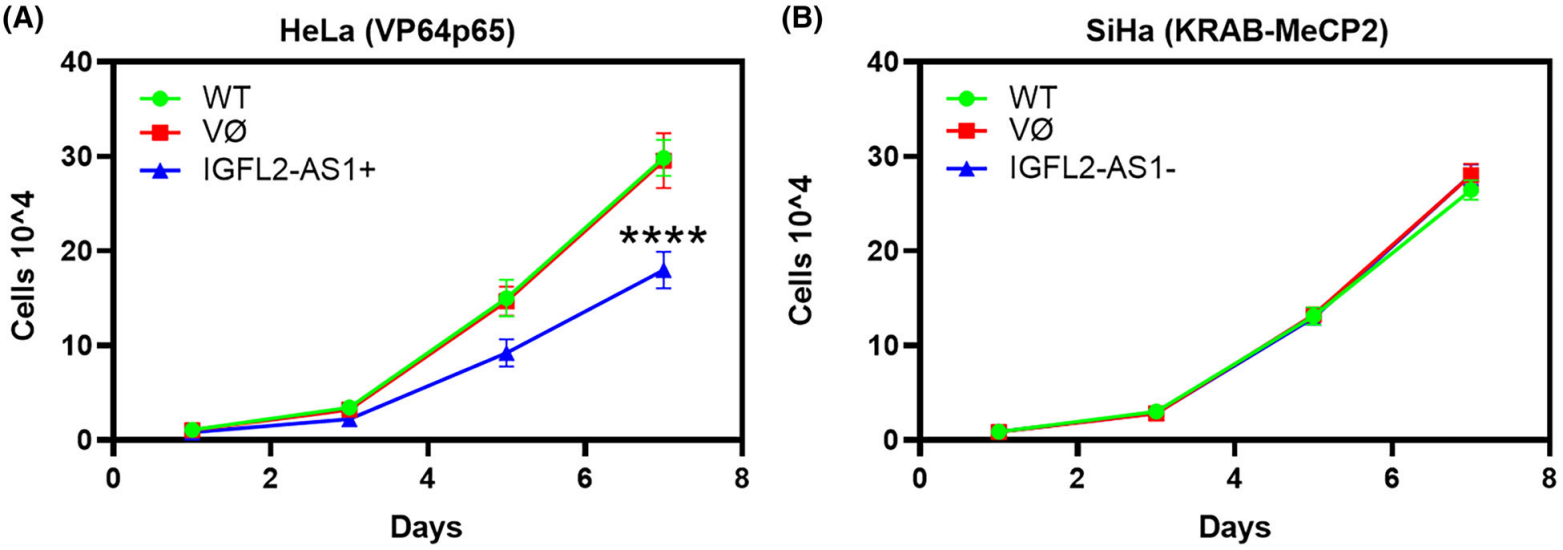
Proliferation curves of cervical cancer cell lines. (A) Growth curves for the adenocarcinoma model (HeLa cells) comparing the proliferation of parental (WT), empty vector‐transduced (VØ), and *IGFL2‐AS1* overexpressing (*IGFL2‐AS1* ^+^) cells over a 7‐day period. (B) Growth curves for the squamous cell carcinoma model (SiHa cells) comparing the proliferation of parental (WT), empty vector‐transduced (VØ), and *IGFL2‐AS1* repressed (*IGFL2‐AS1* ^−^) cells over a 7‐day period. Error bars indicate mean ± SD from three independent experiments. Statistical comparisons between experimental groups and the empty vector control (VØ) were performed using repeated‐measures two‐way ANOVA followed by Dunnett's multiple comparisons test and the corresponding *P*‐values are reported in the figures indicated by ****P < 0.0001. SD: standard deviation.

In HeLa cells, *IGFL2‐AS1* overexpression resulted in a marked reduction in cell proliferation, as evidenced by growth curves over a 7‐day period (Fig. 4A). This effect was specific to *IGFL2‐AS1* overexpression, with these cells exhibiting a consistently lower proliferation rate compared to both parental HeLa cells and empty vector controls (*P* < 0.001). In contrast, downregulation of *IGFL2‐AS1* in SiHa cells did not elicit a change in proliferation rates (Fig. 4B). In fact, growth curves for parental SiHa cells, cells transduced with the empty vector control, and cells with repressed *IGFL2‐AS1* expression were largely overlapping, indicating that reduced *IGFL2‐AS1* levels did not impact the proliferative capacity of these cells under the conditions tested.

### 3.4 *IGFL2‐AS1* modulation differentially impacts migration and clonogenic capacity in cervical cancer cell lines

**Fig. 5:**
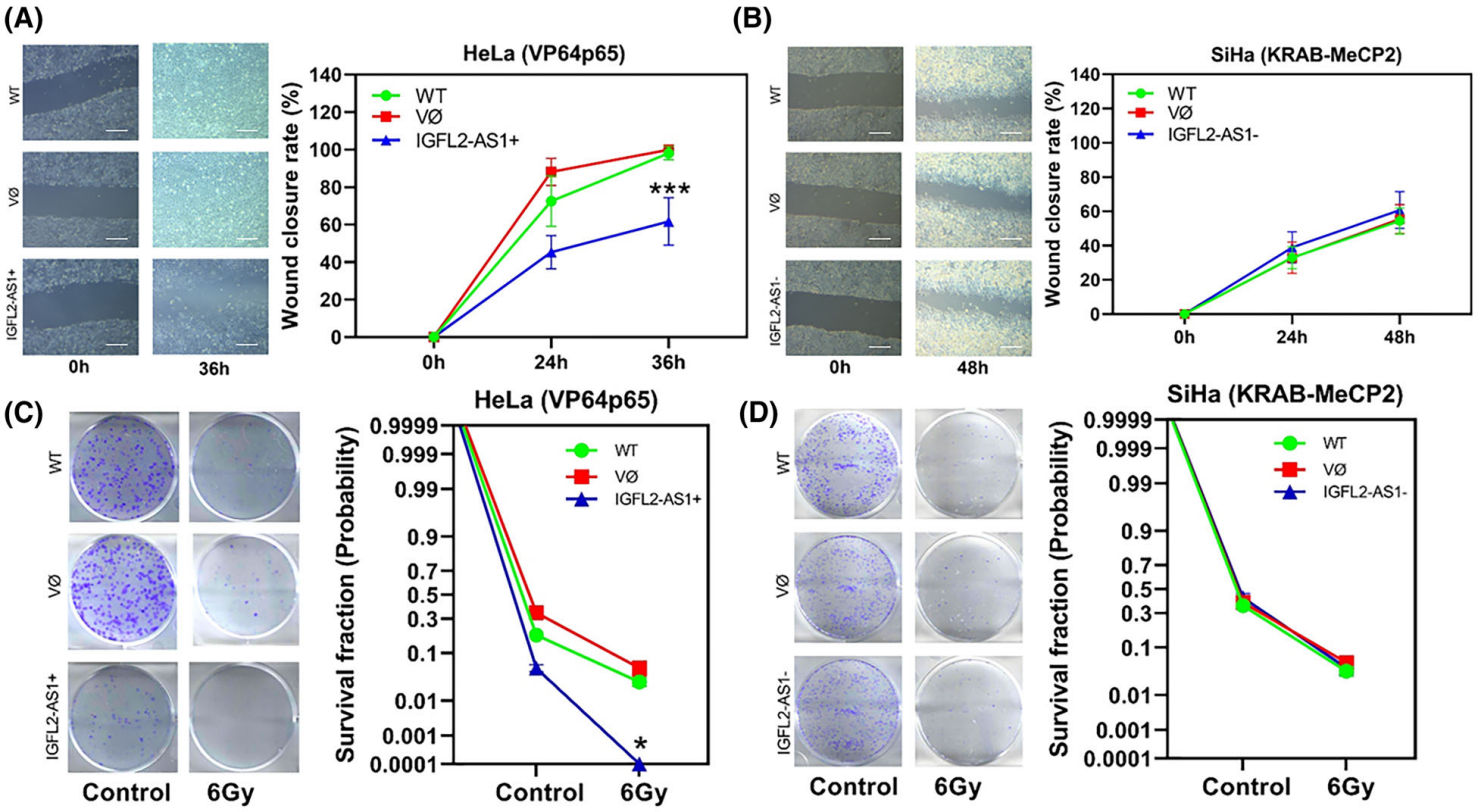
Assessment of migratory and clonogenic capacity following *IGFL2‐AS1* modulation in cervical tumor cells. (A) Analysis of wound closure rate in the wound healing assay for the adenocarcinoma model (HeLa cells) with *IGFL2‐AS1* overexpression (Scale bar: 200 μm). (B) Analysis of wound closure rate in the wound healing assay for the squamous cell carcinoma model (SiHa cells) with *IGFL2‐AS1* repression (Scale bar: 200 μm). (C) Clonogenic assay evaluating colony formation in the adenocarcinoma model (HeLa cells) with *IGFL2‐AS1* overexpression, cultured in the absence (control) or presence of ionizing radiation (6Gy). (D) Clonogenic assay evaluating colony formation in the squamous cell carcinoma model (SiHa cells) with *IGFL2‐AS1* repression, cultured in the absence (control) or presence of ionizing radiation (6Gy). Values represent mean ± standard error of the mean (SEM) from three independent experiments. Comparisons were performed using two‐way ANOVA followed by Dunnett's multiple comparisons test, with statistical comparison conducted relative to the empty vector group (VØ). Statistical significance is indicated by: *P < 0.01, ***P < 0.0001.

We also analyzed the impact of *IGFL2‐AS1* modulation on migration and clonogenic potential. In HeLa cells, *IGFL2‐AS1* overexpression impaired the wound healing capacity, as evidenced by the persistently higher percentage of open wound area observed at both 24‐ and 36‐h post‐scratch time points (Fig. 5A). While control groups (WT and VØ) exhibited near‐complete wound closure at 36 h, HeLa cells with *IGFL2‐AS1* overexpression showed a marked reduction in migration rate, with differences compared to WT cells (*P* < 0.01 at 24 h and *P* < 0.0001 at 36 h). This suggests that elevated *IGFL2‐AS1* expression may attenuate the migratory potential of adenocarcinoma cells, a critical aspect of tumor invasiveness and metastatic dissemination. Conversely, in SiHa cells, reducing *IGFL2‐AS1* expression did not appear to significantly impact cellular migration, as the wound closure rates in cells transduced with the experimental vector were comparable to those observed in cells transduced with the empty vector and in parental (wild type) cells at 24‐ and 48‐h post‐scratch (Fig. 5B). These findings underscore a differential impact of *IGFL2‐AS1* modulation on cellular migration in the two CC models, suggesting that the effects of this modulation may vary according to the tumor subtype.

Clonogenic assays revealed that modulating *IGFL2‐AS1* expression has a notable impact on colony‐forming ability under low‐density conditions, particularly following the stress induced by radiation therapy. Overexpression of *IGFL2‐AS1* (*P* = 0.002; Fig. 5C) induced a drastic reduction in colony formation in HeLa cells, demonstrating a significant therapeutic vulnerability, especially after exposure to 6 Gy radiation, where the ability to form colonies was completely abolished. In contrast, repression of *IGFL2‐AS1* in SiHa cells (Fig. 5D) did not result in significant alterations in clonogenic survival, even after irradiation, suggesting a differential response to *IGFL2‐AS1* modulation between the histological subtypes of CC.

### 3.5 Chemosensitivity is enhanced by *IGFL2‐AS1* overexpression in HeLa cells

**Fig. 6:**
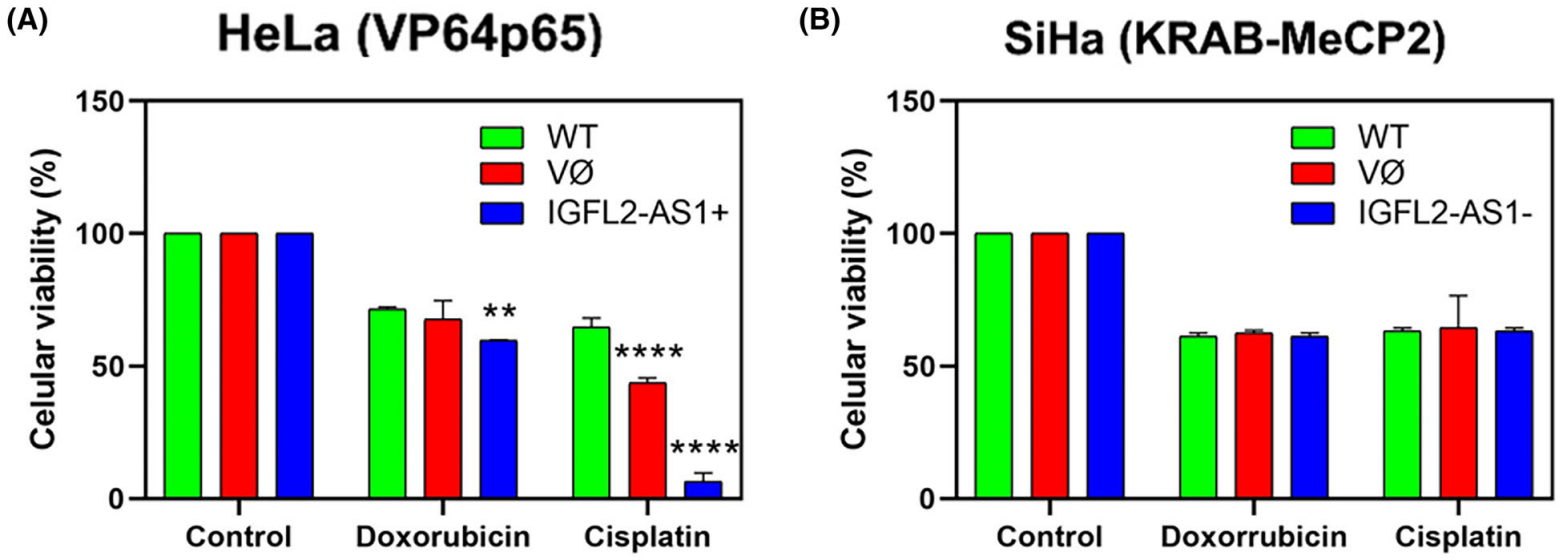
Impact of *IGFL2‐AS1* modulation on cell viability following chemotherapy treatment. (A) Cell viability of HeLa cells after treatment with cisplatin (20 μm) or doxorubicin (0.4 μm) for 72 h. Cells were transduced with either the empty vector (HeLa ∅) or a vector inducing *IGFL2‐AS1* overexpression (HeLa *IGFL2‐AS1* ^+^). (B) Cell viability of SiHa cells after treatment with cisplatin (19 μm) or doxorubicin (0.4 μm) for 72 h. Cells were transduced with either the empty vector (SiHa ∅) or a vector inducing *IGFL2‐AS1* repression (SiHa *IGFL2‐AS1* ^−^). Cell viability was determined using the AlamarBlue™ assay. Data are presented as mean ± standard error of the mean (SEM) from at least three independent experiments. Statistical significance was determined by ANOVA with Tukey's *post‐hoc* test. **P < 0.01, ****P < 0.0001.

The impact of *IGFL2‐AS1* modulation on chemosensitivity was assessed by treating HeLa and SiHa cells with cisplatin or doxorubicin, and subsequently determining cell viability. In HeLa cells (Fig. 6A), *IGFL2‐AS1* overexpression markedly increased sensitivity to cisplatin (*P* < 0.0001), with a drastic reduction in cell viability in *IGFL2‐AS1*‐overexpressing cells. Notably, cells transduced with the empty vector also displayed a reduction in viability following cisplatin treatment. Regarding doxorubicin treatment, no significant difference in cell viability was observed between wild‐type cells and cells transduced with the empty vector (*P* = 0.2527), but *IGFL2‐AS1* overexpression resulted in a small reduction in cell viability (*P* = 0.0015). Conversely, in the SiHa cells, *IGFL2‐AS1* repression did not significantly alter cell viability after treatment with either cisplatin or doxorubicin, with no differences observed across the various treatment groups (Fig. 6B).

**Fig. 7:**
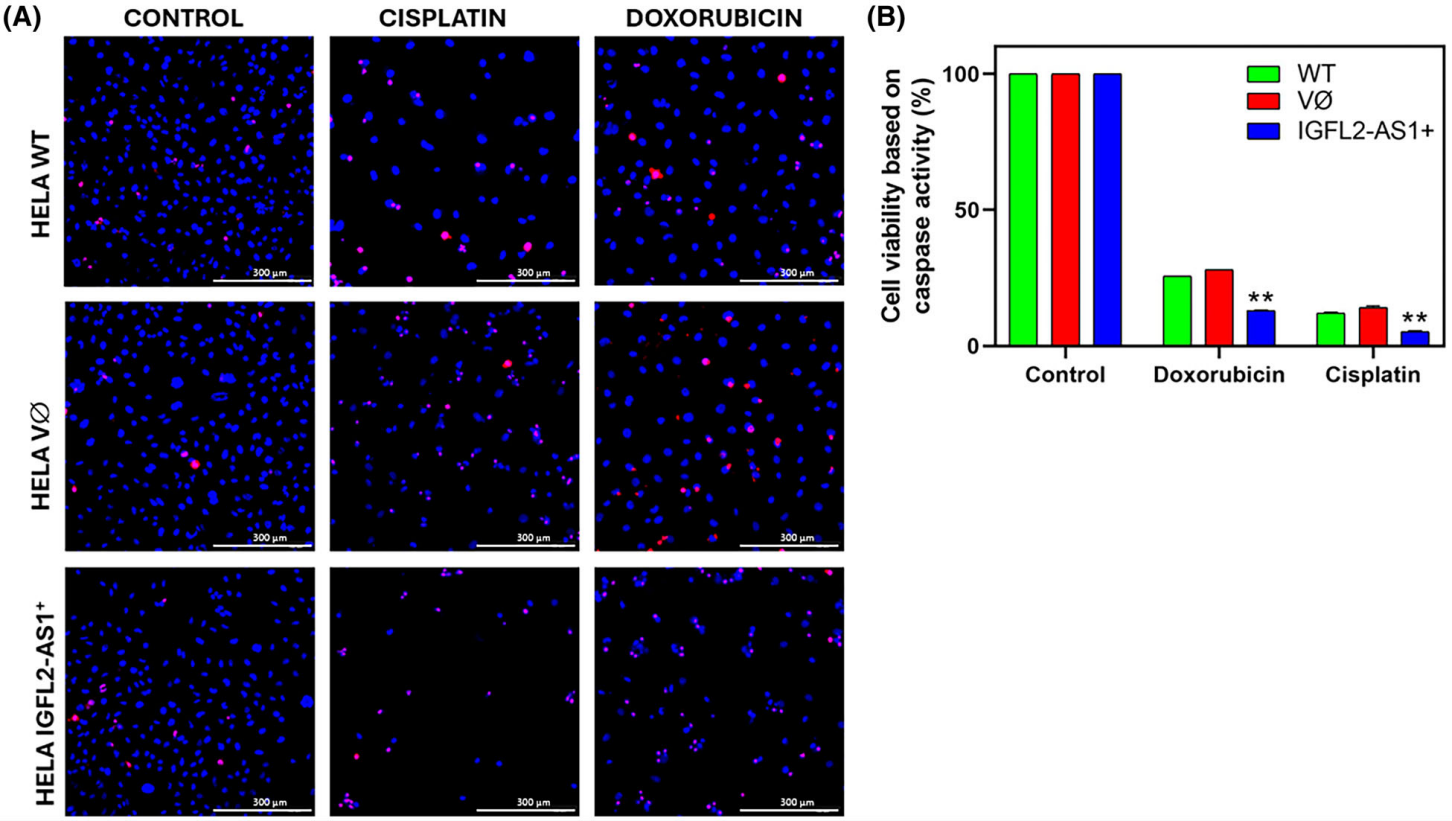
*IGFL2‐AS1* overexpression enhances chemosensitivity and induces apoptosis in HeLa cells. (A) Representative images (×40 magnification) illustrating the impact of *IGFL2‐AS1* overexpression on HeLa cell viability and caspase 3/7 activation after 48 h of cisplatin or doxorubicin treatment. Images reveal reduced cell viability and increased activated caspase 3/7 staining in *IGFL2‐AS1*‐overexpressing cells compared to wild‐type (WT) and empty vector (VØ) controls. Blue: DAPI (nuclei); Red: activated caspase 3/7 activity. Scale bar, 100 μm. (B) Quantitative analysis of overall cell viability in HeLa cells (WT, empty vector, and *IGFL2‐AS1*‐overexpressing) following 48 h of cisplatin or doxorubicin treatment. Viability was calculated from cell adhesion and live cell percentages. *IGFL2‐AS1* overexpression significantly reduced viability after cisplatin and, to a lesser extent, doxorubicin treatment. Data are presented as mean ± standard error of the mean (SEM) from at least three independent experiments. Statistical significance was determined by ANOVA with Tukey's *post hoc* test. **P < 0.01.

To further elucidate the mechanisms underlying the observed reduction in cell viability and enhanced chemosensitivity in HeLa cells, we investigated the activity of caspase 3/7, a critical marker of apoptosis. Visual assessment of treated cells at 48 h revealed reduced cell viability and increased activated caspase 3/7 staining in *IGFL2‐AS1* overexpressing cells (Fig. 7A). Quantitative analysis of overall cell viability demonstrated that *IGFL2‐AS1* overexpression significantly reduced viability after cisplatin treatment (5.25% for *IGFL2‐AS1* ^+^ vs. 14.12% for WT and 12.11% for empty vector) and, to a lesser extent, after doxorubicin treatment (13.52% for *IGFL2‐AS1* ^+^ vs. 29.04% for WT and 26.33% for empty vector) (Fig. 7B).

## 4 Discussion

While global efforts have significantly reduced CC incidence, it persists as a major health challenge, particularly in low‐ and middle‐income countries. A critical aspect of this challenge is the divergent clinical behavior between SCC and ADC, where ADC often presents with a poorer prognosis despite largely similar treatment approaches. Further exacerbating this issue in resource‐limited settings is the high prevalence of HIV co‐infection, which is associated with a disproportionately elevated risk of CC, particularly ADC, compared to the general population [34]. This disparity underscores the urgent need for novel, subtype‐specific biomarkers and therapeutic strategies to improve outcomes for patients with cervical ADC.

LncRNAs can function as either oncogenes or tumor suppressors, depending on the cellular context and the specific repertoire of interacting proteins and other RNAs available within a given cell type [35]. *IGFL2‐AS1* serves as a compelling illustration of this functional complexity. While typically reported as an oncogene in various cancer types exerting its effects through diverse molecular mechanisms, its role appears to differ in CC. For instance, it promotes proliferation, migration, invasion and is linked to radioresistance in colorectal cancer [14], where it acts as a ceRNA by directly binding to miR‐433‐3p, leading to the upregulation of *PAK4* [36]. Similarly, in gastric cancer, it functions as a ceRNA for miR‐802 [15], facilitates progression via the Wnt/β‐catenin pathway in tongue squamous cell carcinoma [18], and confers sunitinib resistance in renal cell carcinoma by directly interacting with heterogeneous nuclear ribonucleoprotein C (hnRNPC) and mediating its transfer via extracellular vesicles [19]. Our group previously reported the differential expression of protein‐coding and noncoding genes in ADC and SCC using RNA‐Seq, and identified the *IGFL2‐AS1* low expression in cervical tumors, especially ADCs [13]. Crucially, in the present study, we further demonstrate that the low expression of *IGFL2‐AS1* is associated with poor overall survival rates in CC patients. This finding is particularly noteworthy as it points toward a tumor‐suppressive role for *IGFL2‐AS1* in CC, contrasting with its reported oncogenic functions in other malignancies.

This observed shift to a tumor‐suppressive role for *IGFL2‐AS1* in CC, contrasting its oncogenic mechanisms in other cancers, underscores its profound context‐dependency. Specifically, we hypothesize that in cervical adenocarcinoma, *IGFL2‐AS1* might exert its suppressive effects through one or a combination of these versatile mechanisms, albeit with altered consequences depending on the cellular milieu. One potential mechanism involves its interaction with hnRNPC, a protein known to promote metastasis. While *IGFL2‐AS1*'s binding to hnRNPC contributes to sunitinib resistance in renal cell carcinoma, in the cervical context, increased *IGFL2‐AS1* expression might instead play a role in attenuating hnRNPC's oncogenic activity (including its regulation of factors like TP53INP2), thereby promoting beneficial outcomes such as apoptosis [19, 37, 38]. Alternatively, or concurrently, *IGFL2‐AS1* could function as a miRNA sponge, similar to its role in colorectal cancer, but targeting different miRNAs and downstream pathways crucial for tumor suppression in the cervical microenvironment [36].

Building on this context‐dependent understanding of lncRNA function, our findings demonstrate that *IGFL2‐AS1* expression and its observed role are distinctly influenced by the cellular environment of different histological subtypes within CC itself. Our previous work had already unveiled distinct transcriptional regulatory networks (TRNs) and differentially expressed lncRNAs, including *IGFL2‐AS1*, characterizing these histological subtypes. Notably, *IGFL2‐AS1* was found to be upregulated in SCC as compared to ADC, and within the SCC TRN, its transcription pattern was linked to the expression of transcription factors (e.g., *FOXN1* and *HES2*), highlighting divergent regulatory mechanisms [13]. Expanding on this, our *in silico* analysis indicated lower *IGFL2‐AS1* expression in HPV18‐associated tumors compared to HPV16 (Fig. S2A). However, this observation requires careful contextualization. The small sample size of HPV18‐positive cases (*n* = 27) in the TCGA‐CESC cohort poses a challenge for interpreting the results, preventing isolation of HPV type as the sole determinant. Crucially, within the ADC subtype, the median *IGFL2‐AS1* expression in HPV16‐positive samples (*n* = 17) was even lower than in HPV18‐positive samples (TCGA dataset, data not shown). This compelling evidence suggests that a specific HPV type may not be the primary driver of *IGFL2‐AS1* expression. Instead, our comprehensive analyses consistently indicate that its differential expression is predominantly determined by the histological subtype. Indeed, ADC samples exhibit virtually no *IGFL2‐AS1* expression, a significant difference from SCC samples, regardless of HPV type—a finding that strongly reinforces its prognostic potential.

This profound influence of histological context is further evidenced by the differential modulation of *IGFL2‐AS1* with epigenetic treatments in cell line models (HeLa and SiHa). The utilization of 5‐AzadC and TSA revealed a striking differential responsiveness of *IGFL2‐AS1* expression between these subtypes. In HeLa cells, 5‐AzadC significantly upregulated *IGFL2‐AS1*, suggesting epigenetic suppression contributes to its low basal expression in this aggressive subtype. Conversely, SiHa cells exhibited distinct response patterns to both 5‐AzadC and TSA, indicating fundamental epigenetic control mechanisms governing *IGFL2‐AS1* expression differ significantly between the histological subtypes. This marked disparity in epigenetic regulation underscores the inherent adaptability of lncRNAs, demonstrating a functional plasticity by acting as either a tumor suppressor or an oncogene depending on the cellular context. This highlights *IGFL2‐AS1* as a potential component in subtype‐specific therapeutic strategies for cervical ADC. Such context‐dependent behavior is further exemplified by *IGFL2‐AS1*'s varied roles in breast cancer, where it has been identified as a tumor suppressor in certain progression models [39]. The *IGFL2‐AS1* role shifts to that of an oncogene in basal‐like breast cancer subtypes, promoting cell proliferation and survival by upregulating pro‐tumorigenic factors including *IGFL1* [17].

While the widespread reliance on the HeLa cell line in CC research is a common critique, it remains the only readily available cell line derived from the primary tumor site for our ADC model. Recognizing the potential for phenotypic biases due to extensive manipulation and contamination, our group, with established expertise in HPV‐associated diseases, paid meticulous attention to the certification and maintenance of this cell line, adhering to strict culturing protocols and international guidelines to ensure its integrity and minimize replication failure [40].

The inherent radioresistance of CC cell lines, particularly HeLa and SiHa, presents a significant challenge in interpreting clonogenic assays. The high clonogenic capacity of these lines, often exceeding 60%, and their remarkable resistance to radiation required careful optimization of the radiation dose and cell density to discern the effects of *IGFL2‐AS1* modulation. Under low‐density conditions, clonogenic survival often depends on autocrine and paracrine signaling, mechanisms that may be compromised during immortalization and cell transformation processes [41]. However, to ensure the robustness of the results, it was established a rigorous criterion for colony counting, defining that only cell aggregates resulting from three to four cell doublings (approximately 10–14 days after plating) were considered viable colonies. The complete absence of viable colonies in HeLa cells overexpressing *IGFL2‐AS1*, especially after irradiation, suggests that restoring *IGFL2‐AS1* expression could potentially influence cellular signaling pathways, making these cells more susceptible to radiation‐induced damage. This finding is particularly relevant considering the well‐established fact that ADCs exhibit greater aggressiveness, invasiveness, and treatment resistance compared to SCCs [8]. Therefore, *IGFL2‐AS1* modulation may represent a promising strategy to sensitize ADC cells to radiotherapy, improving clinical outcomes for this histological subtype.

Extending beyond its role in radiosensitivity, the current investigation also reveals a compelling and differential interplay between *IGFL2‐AS1* expression and the chemo/radiosensitivity of CC cell lines, notably distinguishing between ADC and SCC models. Specifically, we observed that HeLa cells exhibited a substantially higher induction of *IGFL2‐AS1* expression in response to cisplatin, doxorubicin, and irradiation compared to SCC cells. This pronounced upregulation in ADC cells directly correlated with a significant enhancement in their sensitivity to these chemotherapeutic agents following *IGFL2‐AS1* overexpression, as evidenced by marked reductions in cell viability.

Beyond sensitizing ADC cells to conventional therapies, our findings demonstrate that *IGFL2‐AS1* overexpression broadly suppresses the malignant phenotype in this subtype. We observed significantly reduced cell proliferation, diminished cellular migration, and a drastic impairment of clonogenic capacity in HeLa cells upon *IGFL2‐AS1* overexpression, particularly after irradiation. Given that cell proliferation, migration, and clonogenicity are pivotal components of cancer progression, our observations suggest that increasing *IGFL2‐AS1* expression can powerfully mitigate tumor aggressiveness by directly reducing the ability of tumor cells to invade surrounding tissues and subsequently metastasize to distant sites.

Conversely, despite a modest increase in *IGFL2‐AS1* expression in SCC cells upon treatment, its modulation did not translate into significant phenotypic changes. This differential responsiveness strongly suggests that the biological impact of *IGFL2‐AS1* is context‐dependent and highly specific to the ADC subtype, where its expression appears to play a critical role in dictating sensitivity to conventional treatments. Our results suggest that other variables, such as protein‐coding or noncoding genes, may be influencing *IGFL2‐AS1*'s role in SCC. Moreover, the observations may be cell line‐dependent, which requires further confirmation.

These findings lead us to speculate that the minimal basal expression of *IGFL2‐AS1* in wild‐type HeLa cells is insufficient to exert a significant tumor‐suppressive effect in ADC. However, overexpressing *IGFL2‐AS1*, particularly in conjunction with treatment with chemotherapeutic agents like cisplatin, can potentiate its therapeutic effects. Conversely, in SiHa cells, where basal *IGFL2‐AS1* expression is already higher, the cell population may have undergone adaptive changes in signaling pathways that confer radio/chemoresistance, as inferred from the limited variation in *IGFL2‐AS1* expression in response to treatment.

The complex molecular mechanisms underlying cisplatin resistance in CC, including alterations in DNA repair, apoptosis, and epithelial–mesenchymal transition (EMT), have been extensively studied. It is further emphasized that chemoresistance often exhibits a multi‐factorial nature, requiring combination therapies targeting multiple mechanisms to sufficiently enhance cisplatin sensitivity [42]. Furthermore, the less pronounced efficacy of bevacizumab with TP (Paclitaxel and Platinum) therapy in ADC compared to SCC highlights the need for new therapeutic directions in treating cervical ADC, especially in advanced stages, suggesting the involvement of alternative molecular pathways [43].

Considering that CC incidence and mortality disproportionately affect low‐ and middle‐income countries, where access to gold‐standard chemotherapeutic regimens may be limited, strategies that enhance the sensitivity of ADC to more readily available and cost‐effective agents such as cisplatin could prove particularly advantageous. In this context, the mechanistic insights derived from caspase 3/7 activity provide a key mechanistic explanation for this enhanced sensitivity. The observed increase in caspase 3/7 activity in *IGFL2‐AS1*‐overexpressing HeLa cells following treatment with cisplatin or doxorubicin demonstrates that the lncRNA's role in sensitizing ADC cells to chemotherapy is, at least in part, mediated through the activation of apoptosis. This apoptotic induction not only underpins the improved therapeutic response seen in our ADC model but also reinforces *IGFL2‐AS1*'s potential to overcome resistance mechanisms and significantly improve treatment efficacy.

In this study, we effectively leveraged the versatility of CRISPR/dCas9 technology to precisely modulate *IGFL2‐AS1* expression in CC cell models, revealing its context‐dependent role. Overexpression of *IGFL2‐AS1* in ADC‐derived cells reversed aggressive phenotypes, indicating a tumor‐suppressive role. Conversely, the repression of *IGFL2‐AS1* in SCC cells did not result in significant phenotypic alterations, suggesting that low *IGFL2‐AS1* expression may be a poor prognostic factor specifically for ADC, but not a determinant of tumor aggressiveness in SCC. These findings underscore the importance of considering tumor heterogeneity and the specificity of the cellular context when exploring therapeutic targets in cancer. This precise transcriptional control, achieved without altering the DNA sequence and with reduced off‐target effects, highlights the potential of such CRISPR/dCas9 tools for validating novel therapeutic targets. Their versatility, which enables strategies such as activating tumor suppressor genes or silencing oncogenes, as well as resistance mechanisms, paves the way for more effective and personalized therapies in precision oncology [44, 45, 46].

## 5 Conclusions

We demonstrate that *IGFL2‐AS1* plays a context‐dependent role in CC, with distinct functions in ADC and SCC‐derived cell lines. The overexpression of *IGFL2‐AS1* in ADC cells led to a reversal of aggressive phenotypes and increased sensitivity to chemotherapy. Conversely, the repression of *IGFL2‐AS1* in SCC cells did not result in phenotypic alterations, suggesting that low *IGFL2‐AS1* expression may be a poor prognostic factor specifically for ADC, but not necessarily a determinant of tumor aggressiveness in SCC. These findings underscore the importance of considering tumor heterogeneity and the specific molecular characteristics of each histological subtype when developing therapeutic strategies for CC. Furthermore, our data suggest that *IGFL2‐AS1* may be a promising therapeutic target for ADC, particularly in combination with conventional chemotherapeutic agents. However, further studies are needed to fully elucidate the mechanisms by which *IGFL2‐AS1* exerts its effects and to validate its therapeutic potential *in vivo*. Ultimately, our research, by identifying *IGFL2‐AS1* as a histological subtype‐specific biomarker (with low expression indicating poor prognosis) and therapeutic target, contributes to the growing body of evidence supporting the development of personalized approaches for the CC treatment, with the aim of improving outcomes for all patients.

## Conflict of interest

The authors declare no conflict of interest.

## Author contributions

RCC: conceptualization, literature review, methodology, writing, revision, *in vitro* experiments. LPH: *in vitro* experiments and literature review. DRB: writing, statistics, revision, *in silico* analysis. LS: writing, revision. PSAS: writing, revision. FP: writing, revision. LLV: conceptualization, methodology, writing, revision.

## Supporting information


**Fig. S1.** *IGFL2‐AS1* Design and targeting of sgRNAs for CRISPR/dCas9‐mediated modulation of *IGFL2‐AS1* expression. Design and targeting of sgRNAs for CRISPR/dCas9‐mediated modulation of *IGFL2‐AS1* expression. (A) Schematic representation of the *IGFL2‐AS1* gene locus on chromosome 19, indicating its anti‐sense orientation. The positions of the single guide RNAs (sgRNAs) relative to the transcription start site (TSS) are shown (−107 and − 161 bp, respectively). These sgRNAs were employed for both the synergistic activation mediator (SAM‐Cas9) system for transcriptional activation and the KRAB‐MeCP2 system for transcriptional repression. (B) Sequences of the sgRNAs (Top and Bottom strands) designed and synthesized for targeting *IGFL2‐AS1*. Adaptor regions for cloning into the expression vector are highlighted in red.

**Fig. S2.** Additional *in silico* characterization of *IGFL2‐AS1* expression and survival in cervical cancer. Additional *in silico* characterization of *IGFL2‐AS1* expression and survival in cervical cancer. (A) Expression levels of *IGFL2‐AS1* in cervical cancer samples stratified by HPV type (HPV16, n = 103; HPV18, n = 27), independent of histological subtype. Data are presented as box‐and‐whisker plots (Tukey method), where horizontal lines represent the median and quartiles, and whiskers indicate distribution dispersion. Comparisons were conducted using the Mann–Whitney test. (B) Overall survival of cervical adenocarcinoma patients according to *IGFL2‐AS1* expression categorized as low or high based on the median expression of this gene. Censored data are indicated by individual marks on survival curves. Statistical analysis was done using the log‐rank test, and Cox regression was applied for estimation of hazard ratio and confidence interval. Data were obtained from the TCGA and GDC databases.

## Data Availability

The data that support the findings of this study are openly available from The Cancer Genome Atlas (TCGA) and Genomic Data Commons (GDC) via the cBioPortal for Cancer Genomics at www.cbioportal.org, utilizing the TCGA‐CESC cohort.
