# Supplementary figures and images for: Targeted modulation of IGFL2‐AS1 reveals its translational potential in cervical adenocarcinoma
Source: Mol Oncol. 2026 Feb 3;20(6):1643–60. doi: 10.1002/1878-0261.70217 (PMC13238864; doi:10.1002/1878-0261.70217)

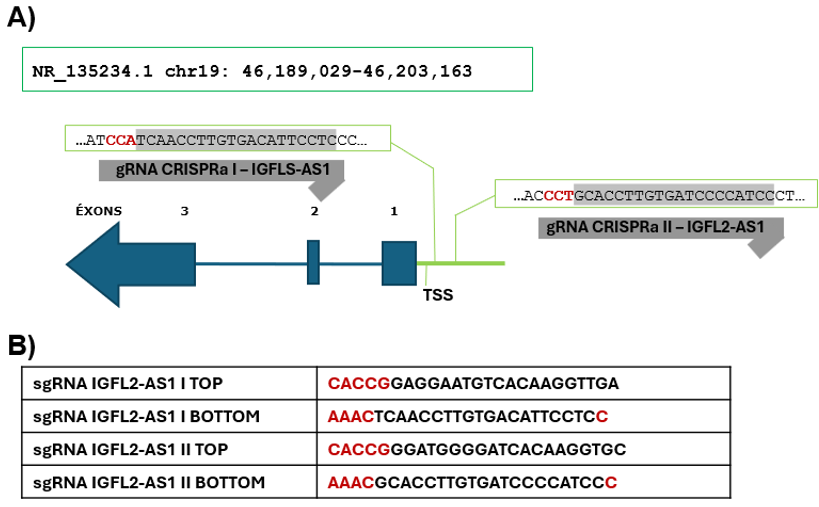

Supplement: Supplementary file 1 — Fig. S1. IGFL2‐AS1 Design and targeting of sgRNAs for CRISPR/dCas9‐mediated modulation of IGFL2‐AS1 expression. Design and targeting of sgRNAs for CRISPR/dCas9‐mediated modulation of IGFL2‐AS1 expression. (A) Schematic representation of the IGFL2‐AS1 gene locus on chromosome 19, indicating its anti‐sense orientation. The positions of the single guide RNAs (sgRNAs) relative to the transcription start site (TSS) are shown (−107 and − 161 bp, respectively). These sgRNAs were employed for both the synergistic activation mediator (SAM‐Cas9) system for transcriptional activation and the KRAB‐MeCP2 system for transcriptional repression. (B) Sequences of the sgRNAs (Top and Bottom strands) designed and synthesized for targeting IGFL2‐AS1. Adaptor regions for cloning into the expression vector are highlighted in red. [file MOL2-20-1643-s002.tif]

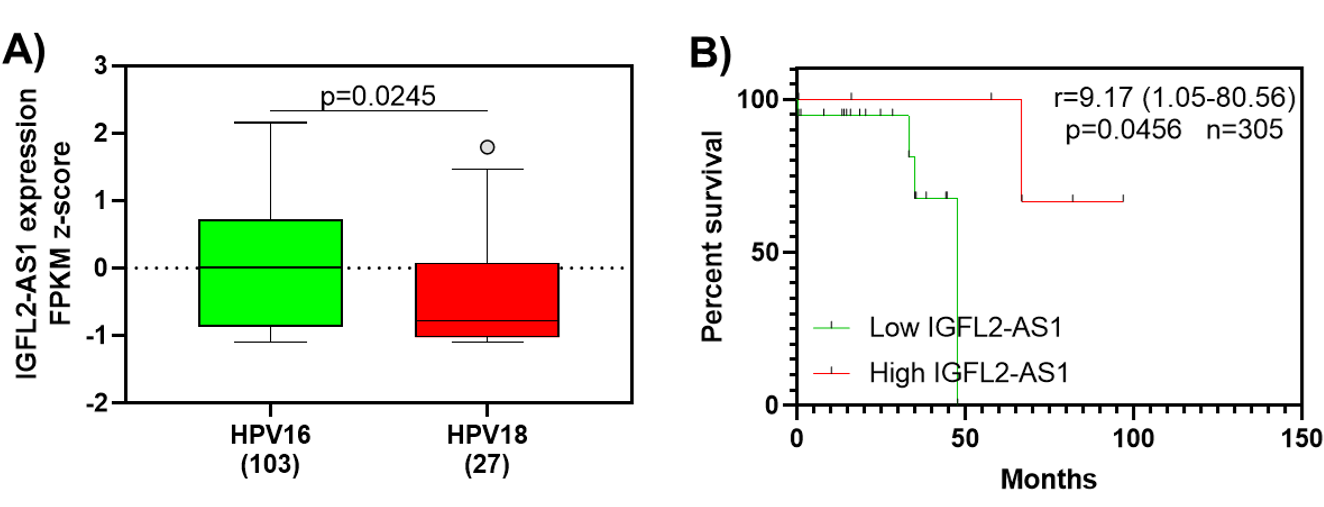

Supplement: Supplementary file 2 — Fig. S2. Additional in silico characterization of IGFL2‐AS1 expression and survival in cervical cancer. Additional in silico characterization of IGFL2‐AS1 expression and survival in cervical cancer. (A) Expression levels of IGFL2‐AS1 in cervical cancer samples stratified by HPV type (HPV16, n = 103; HPV18, n = 27), independent of histological subtype. Data are presented as box‐and‐whisker plots (Tukey method), where horizontal lines represent the median and quartiles, and whiskers indicate distribution dispersion. Comparisons were conducted using the Mann–Whitney test. (B) Overall survival of cervical adenocarcinoma patients according to IGFL2‐AS1 expression categorized as low or high based on the median expression of this gene. Censored data are indicated by individual marks on survival curves. Statistical analysis was done using the log‐rank test, and Cox regression was applied for estimation of hazard ratio and confidence interval. Data were obtained from the TCGA and GDC databases. [file MOL2-20-1643-s001.tif]
